# Supplementary figures and images for: Epithelial cell-turnover ensures robust coordination of tissue growth in Drosophila ribosomal protein mutants
Source: PLoS Genet. 2021 Jan 28;17(1):e1009300. doi: 10.1371/journal.pgen.1009300 (PMC7842893; doi:10.1371/journal.pgen.1009300)

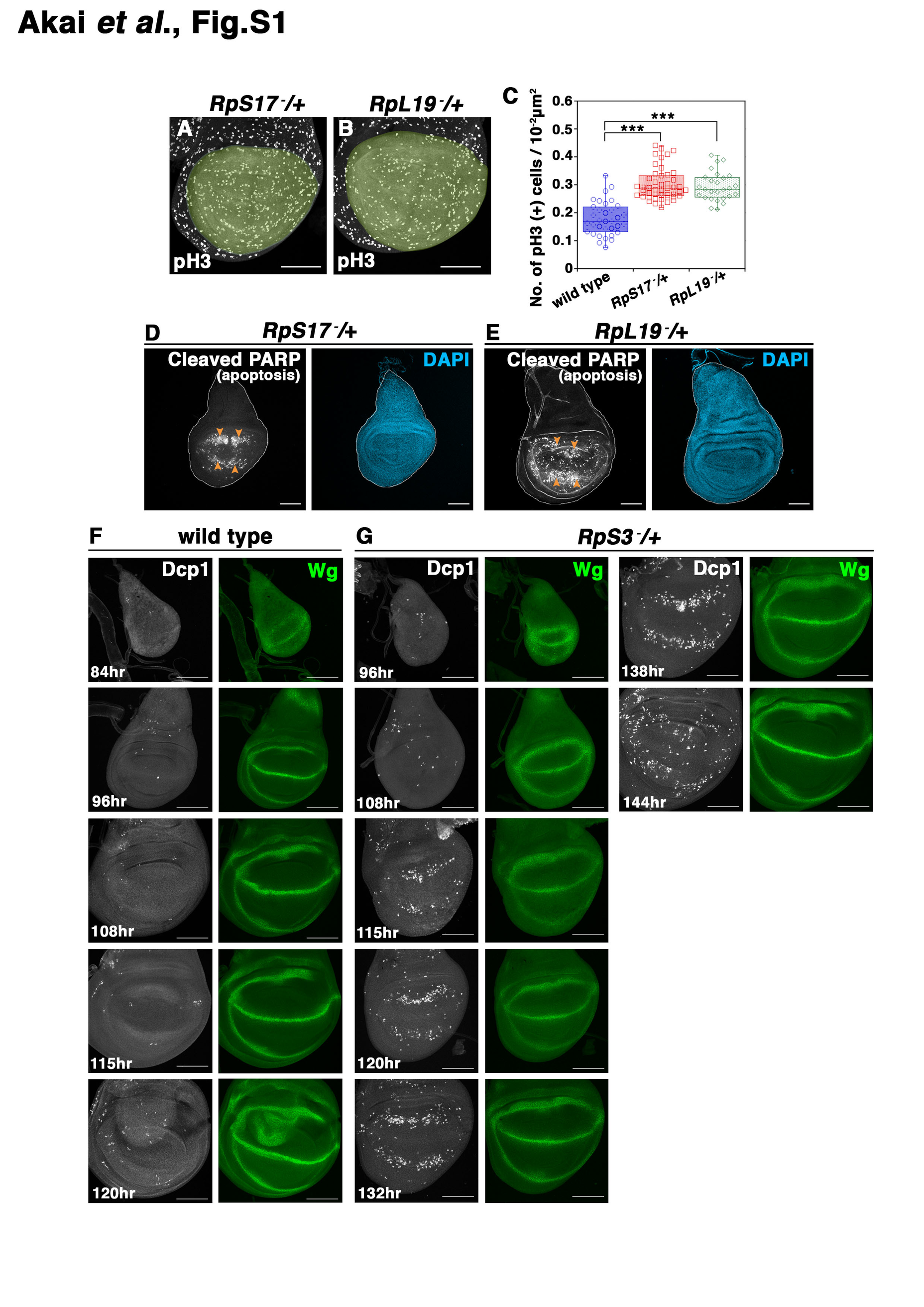

Supplement: S1 Fig — (A and B) Wing disc of RpS17/+ (A) or RpL19/+ (B) flies were stained with anti-phospho-histone H3 (pH3) (Ser10) antibody (white). Wing pouches were marked by pale green. Scale bar, 100 μm. (C) Boxplot with dots representing pH3 positive cells in the pouch in genotypes shown in (Fig 1A) (n = 34, number of wing pouches), (A) (n = 46) and (B) (n = 27). Error bars, SEM; ***, p<0.001; non-parametric Mann-Whitney U-test. (D and E) The activated-caspase-3 indicator CD8-PARP-Venus was expressed in RpS17/+ (D) or RpL19/+ (E) flies and dying cells in the wing disc were visualized by anti-cleaved PARP antibody. The nuclei were visualized by DAPI staining. Arrowheads indicate massive cell death occurred along the D/V axis in the wing pouch. Scale bar, 100 μm. (F and G) Developing wing disc of wild-type or RpS3/+ flies are shown. Dying cells visualized by anti-cleaved Dcp-1 staining (white). Wg expression was visualized by anti-Wg staining (green). Flies were allowed to lay eggs for 2 hours at 25°C on yeast-based food. Larvae were dissected at the indicated time points after egg laying (AEL). All flies were third instar larvae. Scale bar, 100 μm. Genotypes are as follows: nub-Gal4/+; UAS-CD8-PARP-Venus/RpS174 (A, D), nub-Gal4/RpL19K03704; UAS-CD8-PARP-Venus/+ (B, E). SgsΔ3–GFP/+ (F), SgsΔ3–GFP/FRT82B, Ubi-GFP, RpS3Plac92 (G). (TIF) [file pgen.1009300.s001.tif]

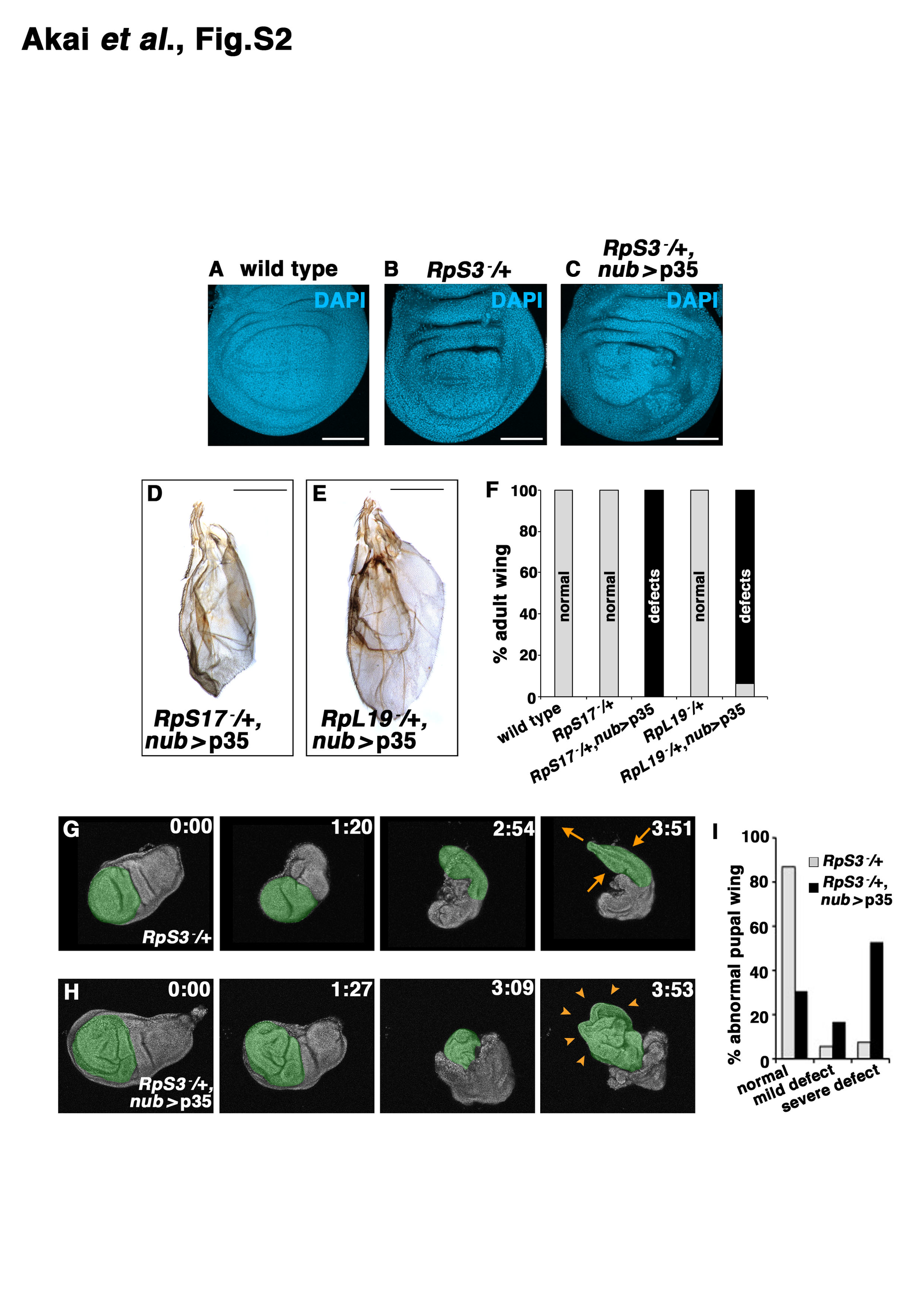

Supplement: S2 Fig — (A-C) Wing discs of wild-type (A), RpS3/+ (B), or RpS3/+, nub-Gal4, UAS-p35 (C) flies. Nuclei were stained with DAPI. Scale bar, 100 μm. (D and E) Adult wings of RpS17/+, nub-Gal4, UAS-p35 (D), or RpL19/+, nub-Gal4, UAS-p35 (E) fly. Scale bar, 500 μm. (F) The rate of defective wings in the genotypes shown in (D) (n = 32) and (E) (n = 31). (G and H) Time-lapse of wing eversion in RpS3/+ (G) or RpS3/+, nub-Gal4, UAS-p35 (H). Arrows indicate the direction of extension. Arrowheads indicate abnormal wrinkled structures. (I) The percentage of abnormal pupal wings in RpS3/+ (n = 53) or RpS3/+, nub-Gal4, UAS-p35 (n = 36), which were classified according to the level of severity. Genotypes are as follows: nub-Gal4/+; UAS-CD8-PARP-Venus/+ (A), nub-Gal4/+; UAS-CD8-PARP-Venus, RpS3Plac92/+ (B), nub-Gal4/+; UAS-CD8-PARP-Venus, RpS3Plac92/ UAS-p35 (C), nub-Gal4/+; UAS-p35/RpS174 (D), nub-Gal4/RpL19K03704; UAS-p35/+ (E), nub-Gal4/+; FRT82B, Ubi-GFP, RpS3Plac92/+ (G), and nub-Gal4/+; FRT82B, Ubi-GFP, RpS3Plac92/UAS-p35 (H). (TIF) [file pgen.1009300.s002.tif]

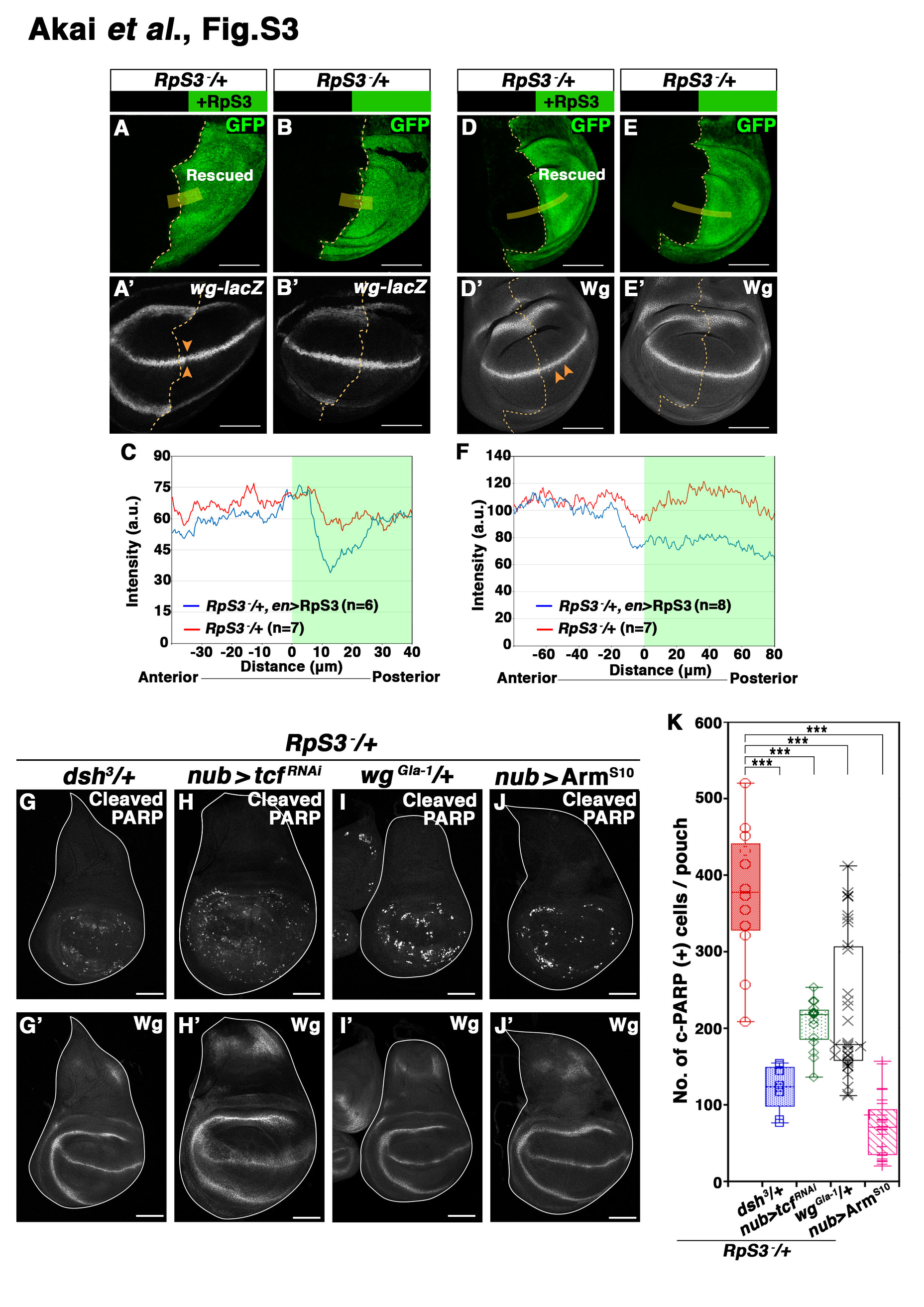

Supplement: S3 Fig — (A-B’) RpS3 was overexpressed in the posterior compartment of wing discs of RpS3/+, wg-lacZ/+ (A) flies using the en-Gal4 driver. wg expression was visualized by anti-β-galactosidase staining (white). Arrowheads indicate relatively lower expression of wg. Scale bar, 100 μm. (C) Quantification of wg intensity in the pale green-marked area shown in (A) (n = 6) or (B) (n = 7). The intensity was automatically or manually measured using ImageJ (Fiji) software as described in the previous manuscript [67]. (D-E’) RpS3 was overexpressed in the posterior compartment of wing discs of RpS3/+ (D) flies using the en-Gal4 driver. Wg expression was visualized by anti-Wg staining (white). Arrowheads indicate relatively lower expression of Wg. Scale bar, 100 μm. (F) Quantification of Wg intensity in the pale green-marked area shown in (D) (n = 8) or (E) (n = 7) are shown. The intensity was automatically or manually measured using ImageJ (Fiji) software as described in the previous manuscript [67]. (G-J) Dying cells were visualized by anti-cleaved PARP staining in RpS3/+, dsh3/+ (G), RpS3/+, nub-Gal4, UAS-tcf-RNAi (H), RpS3/+, wgGla-1/+ (I), or RpS3/+, nub-Gal4, UAS-ArmS10, TG80 ts7(J) wing disc. Massive cell death was significantly suppressed by reducing Wg signal gradient. Scale bar, 100 μm. (K) Boxplot with dots representing cleaved-PARP-positive dying cells per pouch in genotypes shown in (Fig 3B) (n = 12, number of wing pouches), (G) (n = 8), (H) (n = 15), (I) (n = 31), and (J) (n = 24). Error bars, SEM; ***, p<0.001; non-parametric Mann-Whitney U-test. Genotypes are as follows: en-Gal4, UAS-GFP/wg17en40cP1; RpS3Plac92, UAS-RpS3/+ (A), en-Gal4, UAS-GFP/wg17en40cP1; RpS3Plac92/+ (B), en-Gal4, UAS-GFP/+; RpS3Plac92, UAS-RpS3/+ (D), en-Gal4, UAS-GFP/+; RpS3Plac92/+ (E), dsh3/+; nub-Gal4/+; UAS-CD8-PARP-Venus, RpS3Plac92/+ (G), nub-Gal4/UAS-tcf-RNAi; UAS-CD8-PARP-Venus, RpS3Plac92/+ (H), nub-Gal4/wg gla-1; UAS-CD8-PARP-Venus, RpS3Plac92/+ (I), and UAS-ArmS10/+; nub-Gal4/+; UAS-CD8-P [file pgen.1009300.s003.tif]

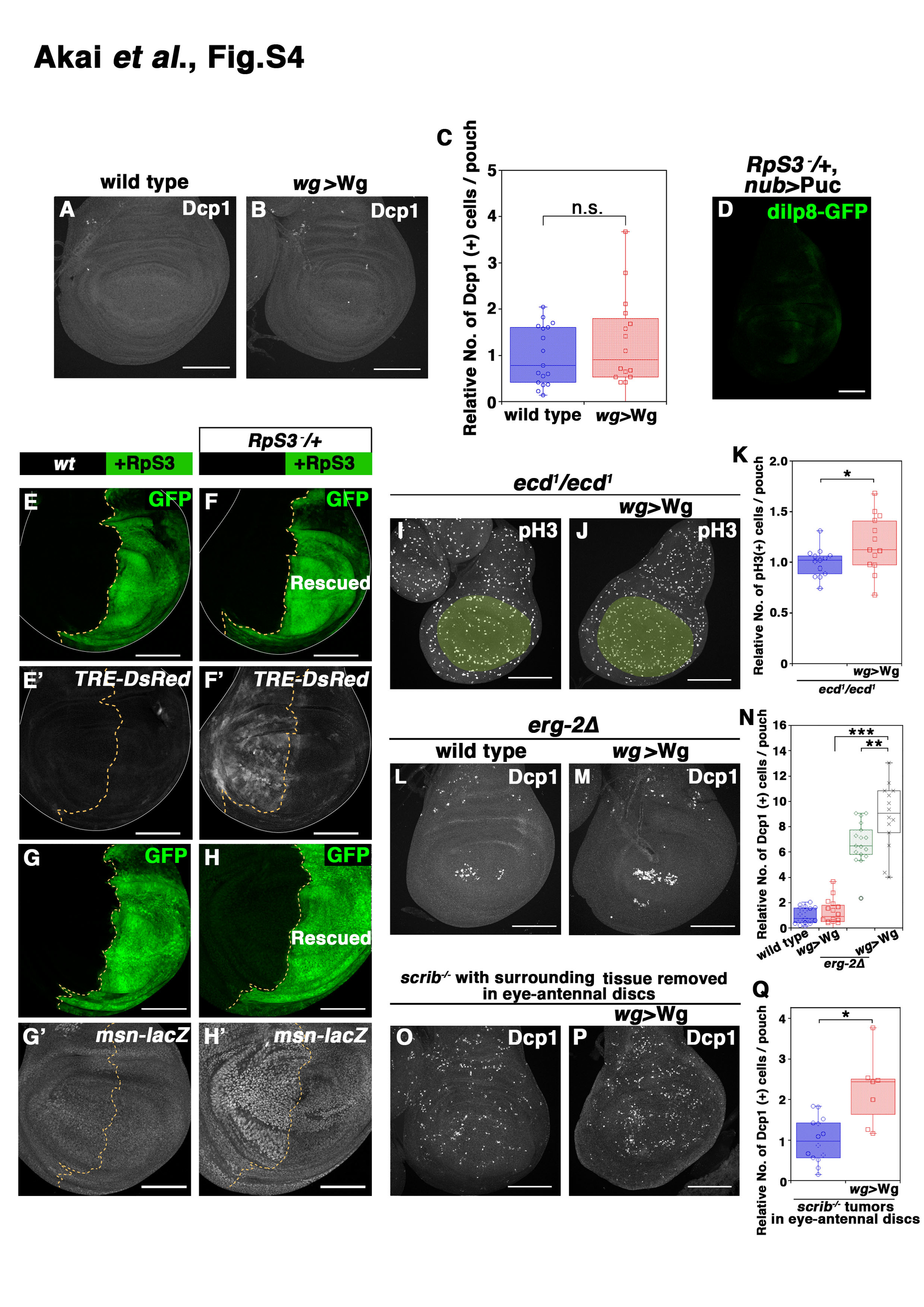

Supplement: S4 Fig — (A and B) Wg was overexpressed using the endogenous wg promoter (wg-Gal4). Dying cells in the wing discs of wild-type (A) or wg-Gal4, UAS-Wg (B) flies were visualized by anti-cleaved Dcp-1 staining. Scale bar, 100 μm. (C) Boxplot with dots representing cleaved-Dcp-1-positive dying cells per wing pouch of genotypes shown in (A) (n = 17, number of wing pouches) and (B) (n = 16). Error bars, SEM; n.s, not significant; non-parametric Mann-Whiteney U-test. (D) The expression of dilp8 in wing discs of RpS3/+, Dilp8::eGFP/+, nub-Gal4, UAS-Puc flies were visualized with GFP (green). Scale bar, 100 μm. (E-F’) RpS3 was overexpressed in the posterior compartment of the wing disc of TRE-DsRed/+ (E) or RpS3/+, TRE-DsRed/+ (F) flies using the en-Gal4 driver. JNK-activated cells were visualized by TRE-DsRed reporter (white). (G-H’) RpS3 was overexpressed in the posterior compartment of the wing discs of msn-lacZ/+ flies (G) or RpS3/+, msn-lacZ/+ (H) flies using the en-Gal4 driver. JNK activity was visualized by anti-β-galactosidase staining (white). Scale bar, 100 μm. (I and J) Wing disc of ecd1/ecd1 (I) or ecd1/ecd1, wg-Gal4, UAS-Wg (J) were stained with anti-phospho-histone H3 (pH3) (Ser10) antibody (white). Wing pouches were marked by pale green. ecd1 is a temperature-sensitive ecd mutant allele that blocks biosynthesis of the active-form of the hormone 20-Hydroxyecdysone at 29°C. For heat-shock treatment, fly culture was transferred to 29°C for 48 hours during the 3rd instar 1arval stage. Scale bar, 100 μm. (K) Boxplot with dots representing pH3 positive cells per pouch in genotypes shown in (I) (n = 13, number of wing pouches) and (J) (n = 13). Error bars, SEM; *, p<0.05; non-parametric Mann-Whitney U-test. (L and M) Wg was overexpressed using the endogenous wg promoter (wg-Gal4) in 3rd instar larvae fed with food containing erg-2 mutant yeast. Dying cells in the wing discs of wild-type (L) or wg-Gal4, UAS-Wg (M) flies were visualized by anti-cleaved Dcp-1 staining. Scale bar [file pgen.1009300.s004.tif]

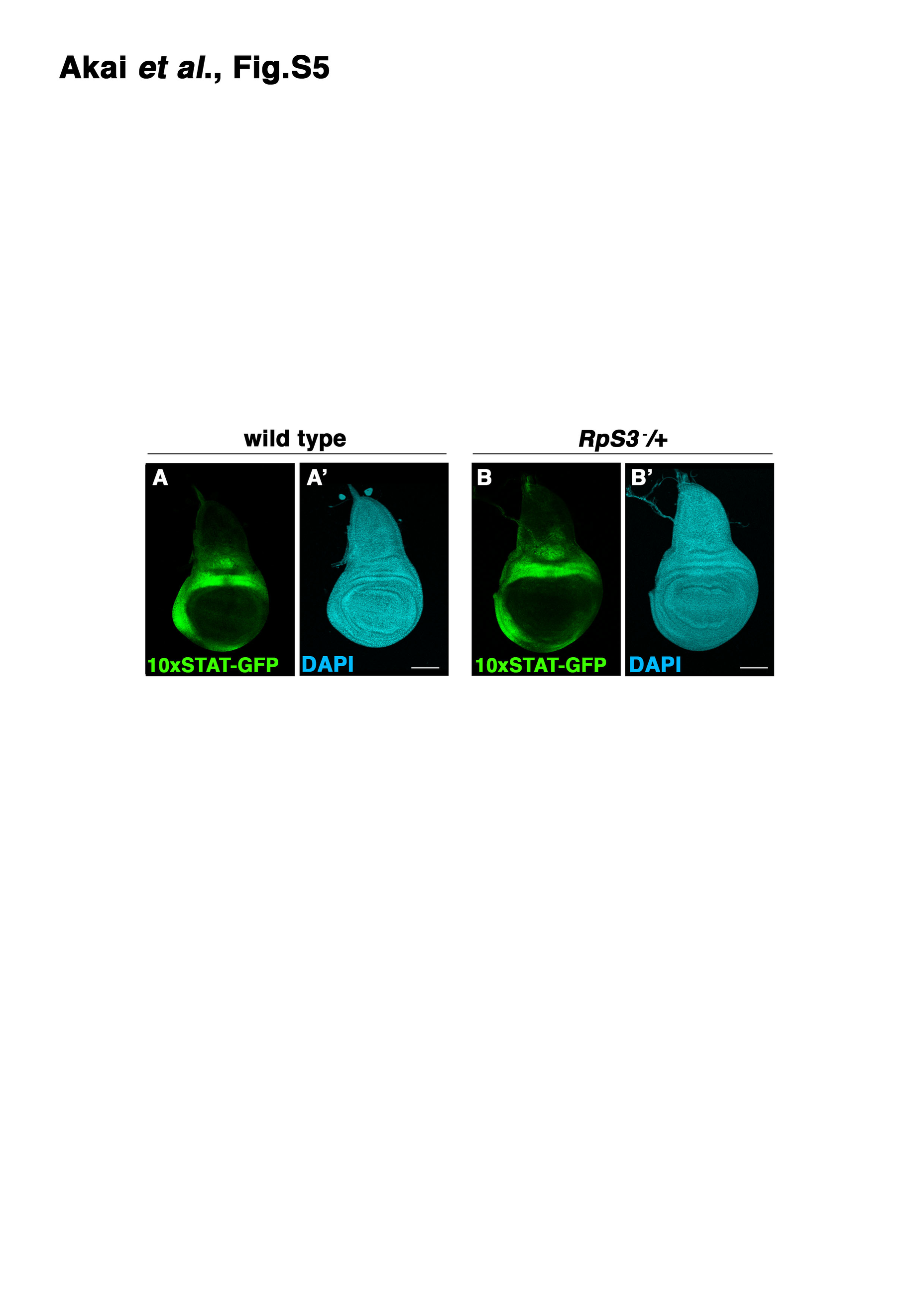

Supplement: S5 Fig — (A-B’) The wild-type (A) or RpS3/+ (B) wing disc bearing the 10xSTAT-GFP reporter (green). The nuclei were visualized by DAPI staining (blue) Scale bar, 100 μm. Genotypes are as follows: 10xSTAT-GFP/+ (A) and 10xSTAT-GFP/ RpS3Plac92 (B). (TIF) [file pgen.1009300.s005.tif]
