# Supplementary material for: Epithelial cell-turnover ensures robust coordination of tissue growth in Drosophila ribosomal protein mutants
Source: PLoS Genet. 2021 Jan 28;17(1):e1009300. doi: 10.1371/journal.pgen.1009300 (PMC7842893; doi:10.1371/journal.pgen.1009300)
Supplement: Attachment — Submitted filename: Response to reviewrs.Akai et al.pdf [file pgen.1009300.s011.pdf]

Responses to Reviewers' comments

We would like to thank the reviewers for helpful comments, which have led us to improve our manuscript substantially. Modifications are indicated in red in the revised manuscript.

Reviewer #1:

This manuscript by Akai et al. reports a study of wing disc development in Minute Drosophila mutant larvae. The authors found abnormally high levels of both cell proliferation and cell death in the wings of these animals. They call this "massive cell turnover". Dissection of this phenomenon showed that it is an instance of regeneration through apoptosis-induced compensatory proliferation, ensuring normal wing development through the known JNK-Wg axis. Furthermore, developmental delay through the also known JNK-Dilp8 axis contributes to the phenomenon. Overall, this is a well-written manuscript reporting a study that is both interesting and expertly conducted, with exquisite quantification of phenotypes. My major concerns have to do with the interpretation of the results, or sometimes lack of it, as I detail below.

1. It is not clear to me what is the ultimate reason that Minute animals show this increased cell death. The model the authors propose is that in Minute/+ animals cell death induces both compensatory proliferation through Wg and developmental delay through Dilp8, and that developmental delay somehow contributes to amplify the response. This is my take from the text. However, the authors are carefully and deliberately ambiguous about the relations among these elements in the model in Fig. 5, grouping proliferation and death together as "cell turnover". "Minute" appears upstream in the diagram, but it is not clear whether this refers to Minute disc or Minute larva. Then, prolonged larval period is predicated of the larva, but it is not clear whether this is the same as the "developmental delay" downstream of Dilp8 or a different, more upstream event. I think a clear possibility is that the whole thing is a consequence of developmental delay and that developmental delay through JNK-Dilp8 is an amplifying event, whereas developmental delay of unknown origin (Dilp8 cannot account for all of it) is the ultimate cause of the whole thing. 4F shows a level of rescue of cell death by ecdysone in the Minute discs that is compatible with this. Furthermore, ecdysoneless mutants in 4M show an amount of cell death that is again compatible with this

possibility. This should be explicitly acknowledged and discussed.

Response:
We thank the reviewer for the comment. As pointed out by the reviewer, developmental delay could be triggered by unknown origin in *M*/+ larvae and is amplified by the JNK-Dilp8 axis, since developmental delay was not fully rescued by blocking the JNK-Dilp8 axis in *M*/+ wing discs. Following the reviewer's suggestion, we have now added the following discussion and made modifications in Fig. 5 (by adding dotted arrows from '*Minute*$^{-}$/+' to 'Developmental delay' and 'Dilp8') in the revised manuscript.

(page 9, paragraph 1)
"The developmental delay in *M*/+ animals could be triggered by unknown origin in *M*/+ larvae and is amplified by the JNK-Dilp8 axis, since developmental delay was not fully rescued by blocking the JNK-Dilp8 axis in *M*/+ wing discs."

2. An alternative interpretation would be that Minute mutant discs have autonomously elevated cell death, intrinsically and independent of developmental delay. In that case, developmental delay induced by cell death would amplify cell death and cell proliferation, but would not be the upstream triggering event. The timing of cell death in Minute discs in Fig S1 suggests that autonomous causes are responsible for elevated cell death and developmental delay later contributes to amplifying it. My understanding is that the field assumes Minute mutant disc cells develop slower but fine. This I thought was the reason the authors repeatedly state that their results are "surprising", but do not explain what is surprising about this. Do the authors agree? Is there anything in their data or in the literature suggesting that Minute cells die at a higher rate in a context-independent way? If that is the interpretation of the authors, there are important consequences for the field and the concept of cell competition itself that the authors should again discuss.

Response:
We thank the reviewer for the comment. We agree that the alternative interpretation can also be possible. However, our data cannot distinguish the possibilities at present and thus we will try to understand it in the future studies. As for the reason for the "surprising" result, the reviewer is right that it has been assumed that *M*/+ disc cells

develop slower without any defects in cell proliferation and cell death. Therefore, following the reviewer's suggestion, we have now added an explanation for the "surprising" result as follows:

(page 9, paragraph 1)
"The lower growth rate of *M/+* clones implied that *M/+* cells simply have a lower cell division rate without any defects in cell proliferation and cell death. Surprisingly, however, we found that…"

Other comments:

A. Line 260: "Our genetic study of M/+ mutants proposes a novel paradigm…" What happens in these discs seems to me very similar to the rn>egr model of regeneration that Iswar Hariharan's lab established. This should be acknowledged and his papers cited.

Response:
Following the reviewer's suggestion, we have now mentioned that the phenomenon we observed is similar to the process of tissue regeneration and cited the rn>egr regeneration paper (Rachel *et al.*, *Dev Cell.*, 2009) in the revised manuscript as follows:

(page 10, paragraph 2)
"The massive cell-turnover induced in the *M*/+ wing pouch ensures normal wing growth during the abnormally prolonged larval period, possibly by creating a flexible cell death and proliferation platform to adjust cell numbers in the prospective wing blade, just like the process of tissue regeneration (53)."

B. Line 267: "Interestingly, it has been shown that a molecular chaperone heat-shock protein 90 (Hsp90) acts as a 'capacitor' for morphological evolution by buffering genetic variations during animal and plant development (52-54). Thus, our findings in M/+ flies suggest that massive cell-turnover may act as a buffering system for developmental time distortion, which could allow morphological variations."

The results here can be understood in the conceptual framework of regeneration through apoptosis-induced proliferation plus injury-induced developmental delay. I really don't

understand the Hsp90 reference here and the relation with morphological evolution.

Response:

As mentioned by the reviewer, cell death, apoptosis-induced proliferation (cell-turnover), and injury-induced developmental delay are all involved in the process of tissue regeneration. However, interestingly, impairment of cell-turnover in *M/+* animals induced phenotypic variations (Figs 2M and 2N), suggesting that cell-turnover may also act as a buffering system against developmental perturbation, just like Hsp90.

To clarify this point, we have now modified the sentence as follows:

(page 10, paragraph 2)

"The fact that impairment of cell-turnover in *M/+* animals induces phenotypic variations (Figs 2M and 2N) suggests that cell-turnover may provide the flexibility to change developmental programs."

C. The authors in the abstract write "Minute/+ mutants". They should write instead "Minute mutants", later clarifying in the introduction that these are dominant haploinsufficient mutations and that the analysis corresponds to Minute heterozygous (or hemizygous) animals (M/+).

Response:

Following the reviewer's suggestion, we have now written "*Minute* mutants" instead of "*Minute*/+ mutants" in the abstract and then explained in the introduction that we analyzed *Minute* heterozygous animals (*M*/+).

D. Expressions like "surprisingly", "strikingly", "interestingly" or "intriguingly" should be used more sparingly by the authors if they want them to achieve any effect.

Response:

Following the reviewer's comment, we have now deleted many of these expressions as follows:

(page 4, paragraph 2)

“Here, we find ~~surprising~~ evidence that both cell death and compensatory cell proliferation are dramatically increased in the *M*/+ wing imaginal epithelium, which are essential for robust wing development in *M*/+ animals.”

(page 7, paragraph 2)
“In addition, Wg signaling activity was ~~intriguingly~~ elevated much more broadly in the *RpS3*/+ pouch compared to the same-stage wild-type (wondering larval stage), as assessed by the *nmo-lacZ* reporter (31) (Figs 3C and 3D).”

(page 8, paragraph 2)
“~~Intriguingly~~, However, Wg-overexpression using the endogenous *wg* promoter did not induce cell death in the wing pouch (S4A and S4B Figs, quantified in S4C Fig).”

(page 11, paragraph 3)
“~~Interestingly~~, It has been reported that JAK/STAT signaling acts downstream of JNK signaling to coordinate regenerative cell proliferation and developmental delay through upregulating *dilp8* expression during regeneration of the *Drosophila* fragmentated leg imaginal disc (61).”

E. Semi-quantitative words should be avoided:
Massive cell turnover, massive cell death
Line 89: much lower
Line 111: only occasionally
Line 244: slightly increased

Response:
Following the reviewer’s comment, we have now modified these semi-quantitative words as follows:

Line 89 (page 5, paragraph 1 in the revised manuscript): ~~much~~ lower
Line 111 (page 5, paragraph 2 in the revised manuscript): only a few dying cells were ~~only occasionally~~ detected
Line 244 (page 9, paragraph 3 in the revised manuscript): ~~slightly~~ moderately increased

As for the term “massive” cell-turnover or “massive” cell death, we used these expressions together with quantitative data (Figs 1M, 1P, 2I, 3L, 4G, 4K, 4O, S3K, S4N, and S4Q Fig). We believe that this helps readers to quantitatively understand the phenomenon.

F. Others:
Line 180: a phenomenon BY WHICH
Line 214: PARTIALLY suppressed
Line 250: number of DYING CELLS

Response:
We have now corrected these words in the revised manuscript.

Reviewer #2:

The manuscript by Akai et al., provides a detailed molecular genetic analysis of the developmental delay phenotype caused by heterozygosity for ribosomal mutants in Drosophila. These mutations, collectively referred to as Minutes, are known to cause pronounced developmental delay, but with the exception of a thin bristle phenotype, produce largely normal adults. Akai et al., were curious how normally proportioned adults where produced by Minute heterozygous mutants even though they experience substantial developmental delay. In recent years it has been discovered that Minute mutants induce expression of the insulin/relaxin like factor dilp8. Dilp8 is a stress signal that attenuates the production of the steroid hormone ecdysone, the metamorphosis inducing factor, thus prolonging developmental time. Therefore, a simple explanation for the relatively normal adult body size and patterning of Minute mutants might have been that in Minute heterozygous larvae all mitotic cells grow and divide slowly as a result of a reduced ribosome pool. This leads induction of the dilp8 stress signal resulting in delayed metamorphosis. Thus, the slow growth rate is compensated by developmental delay leading to the formation of essential normal adults. What the authors found, however, was something much more intriguing. When they examined the proliferation rates of Minute heterozygous imaginal tissue, they discovered that it was

actually significantly increase rather than decreased as the simple model might predict. Therefore, in order to achieve proper size and pattern, the enhanced proliferation must be compensated by some additional mechanism. The authors found that the compensatory mechanism was dramatic induction of apoptosis. They then go on to investigate the molecular mechanism responsible for the Ying and Yang of proliferation/apoptosis and found that Minute heterozygosity leads to up-regulation of Wingless at the D/V compartment which triggers cell-competition as a result of an altered Wg gradient. Intriguingly, they further found that simply altering the Wg gradient in wildtype background is not sufficient to induce the massive apoptosis but that it also requires the dip8 mediated developmental delay. When they artificially altered the Wg gradient and slowed developmental time in a wt background, then apoptosis was induced. The authors conclude that robust cell turnover helps maintaining tissue homeostasis by interfacing with developmental timing mechanisms.

Overall, I find this to be a remarkable set of observations which leads to many additional questions. Obviously, these could be the subject of future studies, but perhaps a few additional experiments could lend a bit of additional clarity to the results.

The authors investigate JNK signaling as a means of inducing dilp8 but recognize that there must be some additional cues. Several other inputs that regulate dilp8 expression have been identified including Hippo and Jak/Stat signaling, the transcription factor Xrp1 and the Ecdysone Receptor. Although the authors mention that Jak/Stat signaling does not seem to be involved, have the examined the requirement of these other factors and pathways?

Response:
We thank the reviewer for the comment. Following the reviewer's suggestion, we have examined whether inhibition of Yki, Xrp1, or Ecdysone Receptor (EcR) affects *dilp8* expression. Interestingly, we found that, while *yki-RNAi* did not affect *dilp8-GFP* expression, overexpression of *Xrp1-RNAi* or a dominant-negative form of EcR (EcR^{DN}) in the posterior compartment of the *RpS3*/+ wing disc significantly suppressed *dilp8-GFP* expression (see Fig R1, below). However, we also found that JNK activation was blocked in the *Xrp1-RNAi*- or EcR^{DN}-expressing compartment (Fig. R1, see below), suggesting that Xrp1 and EcR are required for JNK activation in *RpS3*/+ wing discs. Thus, we have not been able to find any additional cues, other than JNK signaling, for the induction of dilp8. We would like to investigate this issue in the future studies.

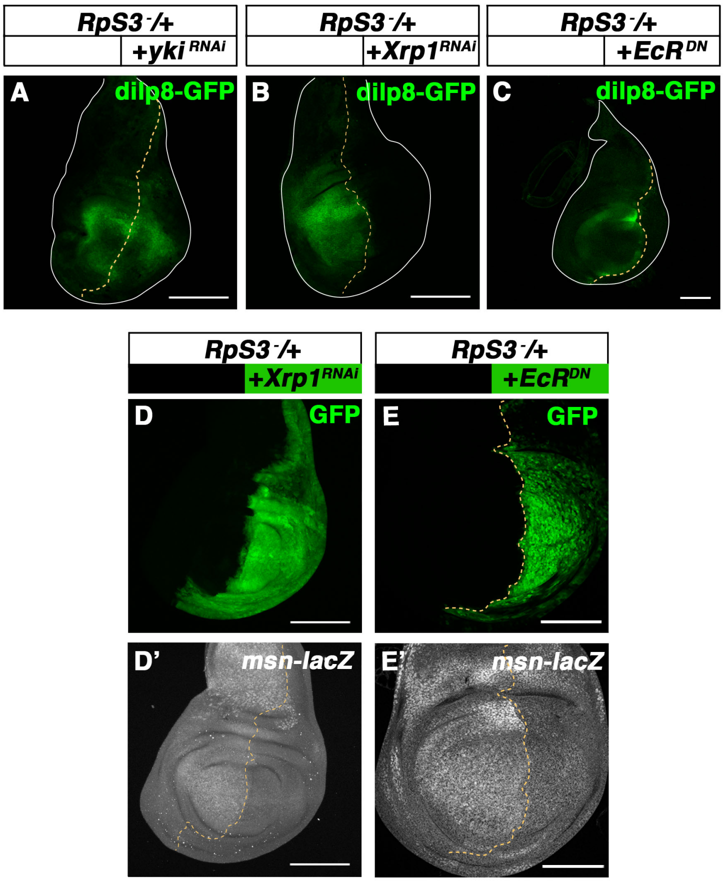

Fig. R1

(A-C) The expression of dilp8 in wing discs of *RpS3/+, dilp8::eGFP/+, en-Gal4, UAS-yki-RNAi* (A), *RpS3/+, dilp8::eGFP/+, en-Gal4, UAS-Xrp1-RNAi* (B), or *RpS3/+, dilp8::eGFP/+, en-Gal4, UAS-EcR-DN* (C) flies were visualized with GFP (green). For heat-shock treatment, fly culture was transferred to 29˚C for 12hours (A) or 48 hours (C) during the 3rd instar 1arval stage. Scale bar, 100 μm.

(D-E') *Xrp1-RNAi* (D) or *EcR-DN* (E) was overexpressed in the posterior compartment of the wing discs of *RpS3/+*, *msn-lacZ/+* flies using the *en-Gal4* driver. JNK activity was visualized by anti-β-galactosidase staining (white). For heat-shock treatment, fly culture was transferred to 29˚C for 24 hours (E) during the 3rd instar larval stage. Scale bar, 100 μm.

Genotypes are as follows: *en-Gal4, UAS-RFP/UAS-yki-RNAi; dilp8::eGFP /*$RpS3^{Plac92}$*.*$TG80^{ts7}$ (A), *en-Gal4, UAS-RFP/UAS-Xrp1-RNAi; dilp8::eGFP/*$RpS3^{Plac92}$ (B), *en-Gal4, UAS-RFP/UAS-EcR-DN; dilp8::eGFP/*$RpS3^{Plac92}$*.*$TG80^{ts7}$ (C), *en-Gal4, UAS-GFP/UAS-Xrp1-RNAi;* msn^{06946}*/*$RpS3^{Plac92}$ (D), and *en-Gal4, UAS-GFP/UAS-EcR-DN;*

msn^{06946}*/ RpS3*^{Plac92}*.TG80*^{ts7} (E).

Another key regulator of imaginal disc growth is Dpp. Did the authors examine Dpp expression to see if it was altered in addition to Wg?

Response:

Following the reviewer's comment, we have examined whether Dpp expression is also altered in *M*/+ wing discs. As a result, we found that *dpp-LacZ* expression was not altered in *M*/+ wing discs, as the expression pattern of *dpp-LacZ* was indistinguishable between *M*/+ and wild-type wing discs overexpressing RpS3 only in the dorsal compartment (genetic rescue of RpS3) (see Fig R2, below).

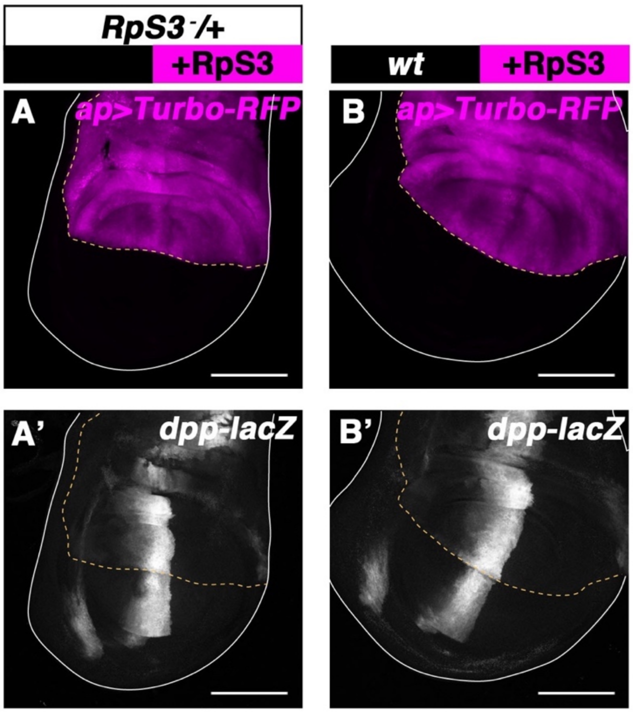

Fig. R2

(A-B') RpS3 was overexpressed in the dorsal compartment of the wing discs of *RpS3*/+ (A) or wild-type (B) flies using the *apterous-Gal4* driver. *dpp* expression was visualized by anti-β-galactosidase staining (white). Scale bar, 100 µm.

Genotypes are as follows: *ap-Gal4/dpp-lacZ; UAS-Turbo-RFP/UAS-RpS3, RpS3*^{Plac92} (A), and *ap-Gal4/dpp-lacZ; UAS-Turbo-RFP/UAS-RpS3* (B).

Another question concerns the degree to which the wing disc response is directly related to developmental time. The authors say it is perturbation of developmental time that is an important aspect of the process and to illustrate this they perturb timing by using the ecd ts mutant while over-expressing Wg in its endogenous pattern which produces strong apoptosis in the wing pouch. But, is it really timing or simply the basal level of ecdysone which is sensed by the disc? Can the authors see the same effect if they block ecdysone reception in the disc itself by either expressing dominant negative EcR or perhaps overexpress RNAi of ECI, the ecdysone importer. This would require using two different conditional expression systems but would add some clarity to the actual mechanism. Likewise, the ecd ts mutant, while convenient, is not the best way to specifically alter E production since it is a splicing factor that may affect many other processes in addition to ecdysone production. Again, the use of two different conditional systems, one to knockdown E production in the PG and the other to overexpress Wg would be cleaner than using the ecd ts mutation.

Response:
We thank the reviewer for the comments. As for the first comment ("Is it really timing or simply the basal level of ecdysone which is sensed by the disc? Can the authors see the same effect if they block ecdysone reception in the disc itself by either expressing dominant negative EcR or perhaps overexpress RNAi of ECI?"), due to a difficulty in performing the suggested experiments using two different conditional expression systems within several months, we focused on the phenotype that ecd ts mutants or wild-type larvae raised in erg-2Δ food show, although not "massive", significantly increased cell death without ectopic overexpression of Wg (Figs 4M and S4L). As a result, we found that blocking ecdysone reception by overexpressing EcR^{DN} or *ECI-RNAi* in wild-type wing discs did not increase (even suppressed) cell death (see Fig R3, below), suggesting that ecdysone level might not be a determinant for the induction of cell-turnover.

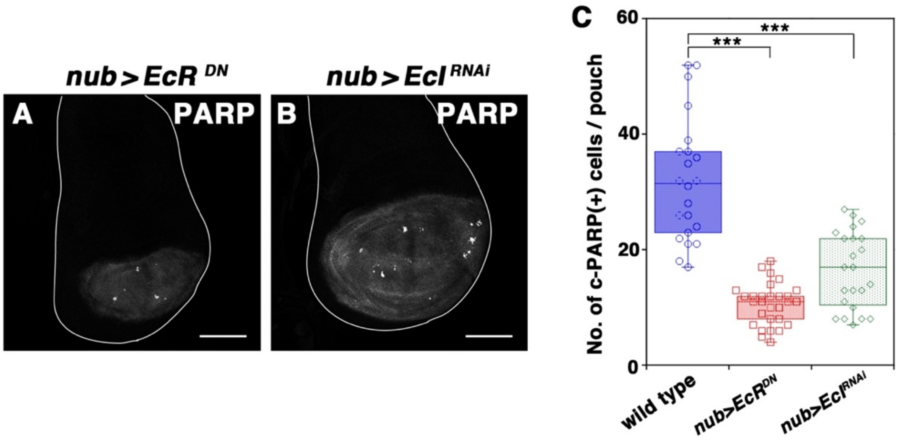

Fig. R3

(A and B) Dying cells were detected by anti-cleaved PARP staining (white) in the wing discs of *nub-Gal4, UAS-EcR-DN* (A), or *nub-Gal4, UAS-EcI-RNAi* (B) flies expressing CD8-PARP-Venus. Scale bar, 100 μm.

(C) Boxplot with dots representing cleaved-PARP-positive dying cells per pouch in genotypes shown in wild-type (n=22) (A) (n=29) (B) (n=23). Error bars, SEM; ***, p<0.001; non-parametric Mann-Whitney *U*-test.

Genotypes are as follows: *nub-Gal4/UAS-EcR-DN; UAS-CD8-PARP-Venus/+* (A), and *nub-Gal4/+; UAS-CD8-PARP-Venus/UAS-EcI-RNAi* (B).

As for the second comment ("The use of two different conditional systems, one to knockdown E production in the PG and the other to overexpress Wg would be cleaner than using the ecd ts mutation"), again due to a difficulty in performing the suggested experiments within several months, we focused on the phenotype that tumor development in the imaginal discs causes developmental delay by inducing dilp8 expression (Colombani *et al*., Science, 2012). As a result, we found that the number of dying cells was moderately increased in the wing discs of animals bearing *scribble*$^{-/-}$ (polarity-deficient) tumors in the eye discs. In addition, a forced increase in Wg expression using the endogenous *wg* promoter in conjunction with developmental delay by *scribble*$^{-/-}$ tumors in the eye discs caused massive cell death in the wing discs. These data support the conclusion that the induction of cell-turnover is due to a developmental delay in conjunction with Wg upregulation.

We have now included these new data in the revised manuscript as Fig S4O-Q as follows:

(page 10, paragraph 1)

"Furthermore, induction of homozygous mutations in an apicobasal polarity gene *scribble* (*scrib*$^{-/-}$) in the entire eye discs, which resulted in the formation of tumors and extended larval period (52), significantly increased the number of dying cells in the wing discs (S4O Fig, compared to S4A Fig, quantified in S4Q Fig). A forced increase in endogenous Wg expression in conjunction with the tumor-induced developmental delay resulted in more increased cell death in the wing pouch (S4P Fig, quantified in S4Q Fig)."

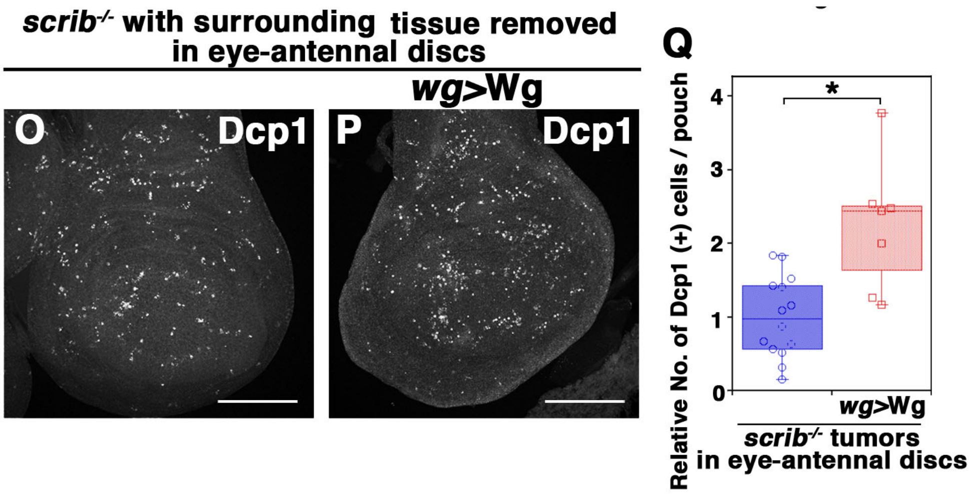

S4 Fig.

(O and P) Dying cells in the wing disc were visualized by anti-Dcp1 antibody staining (white). Wing disc of wild-type (O) or *wg-Gal4,* UAS-Wg (P) shown in flies bearing the eye disc in which *scrib* mutant clones were generated and surrounding wild-type cells were simultaneously removed by a combination of GMR-hid and a recessive cell-lethal mutation, CL3R. Scale bar, 100 μm.

(Q) Boxplot with dots representing cleaved-Dcp-1-positive dying cells per pouch in genotypes shown in (O) (n=14, number of wing pouches), and (P) (n=7). Error bars, SEM; *, $p<0.05$; non-parametric Mann-Whitney *U*-test. Scale bar, 100 μm.

Genotypes are as follows: *eyFLP1/+;; FRT82B,scrib*^{1}*/FRT82B, GMR-hid.CL3R* (O), and *eyFLP1/+*; *wg-Gal4/UAS-Wg*; *FRT82B,scrib*^{1}*/FRT82B, GMR-hid.CL3R* (P).

Another issue is whether heterozygosity for a Minute is only affecting cell turnover in

the imaginal discs. Have the authors examined if other mitotic tissues such as the brain, or mitotic portions of the gut and/or the histoblasts also affected?

Response:

Following the reviewer's suggestion, we have examined whether cell death was increased in other mitotic tissues in *M*/+ animals. As a result, we found that the number of dying cells was not changed in the brain, midgut, and histoblasts in the 3rd instar larvae of *RpS3*/+ animals (see Fig R4, below).

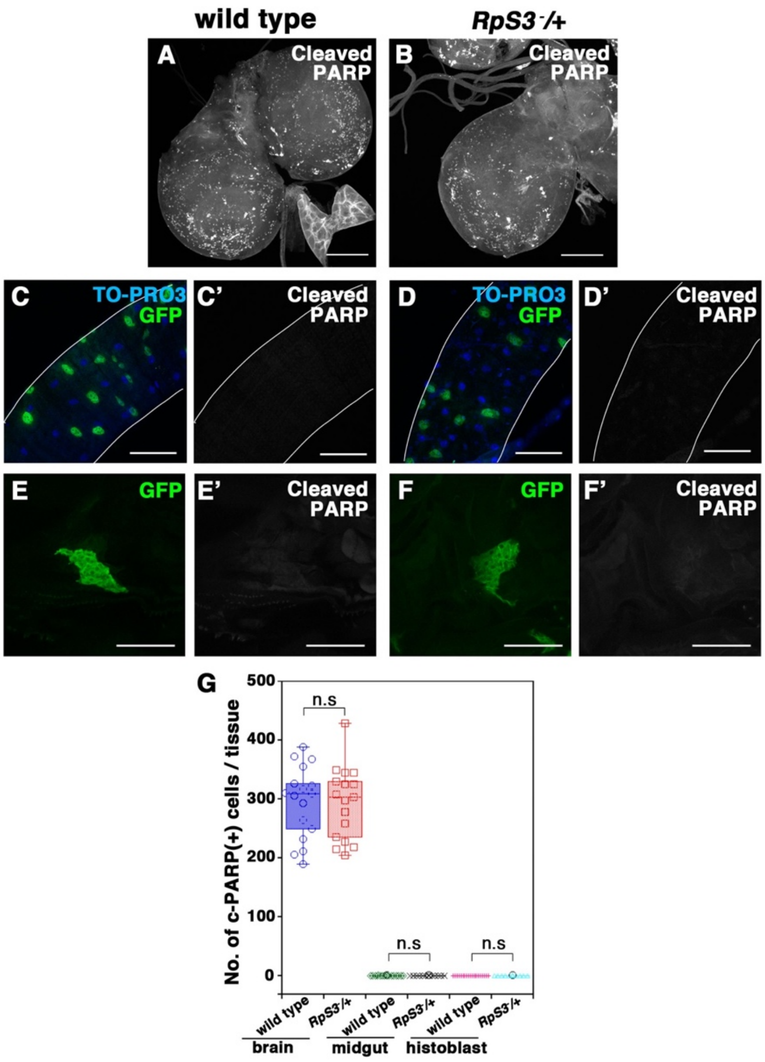

Fig. R4.

(A and B) Dying cells were detected by anti-cleaved PARP staining (white) in the brain of *tub-Gal4*(A), or *RpS3*/+, *tub-Gal4* (B) flies expressing CD8-PARP-Venus. Scale bar, 100 μm.

(C-F) Dying cells were detected by anti-cleaved PARP staining (white) in the midgut (C-D') or histoblast (E-F') of *esg-Gal4* (C, E), or *RpS3*/+, *esg-Gal4* (D, F) flies expressing

CD8-PARP-Venus (green). Scale bar, 100 μm.

(G) Boxplot with dots representing cleaved-PARP-positive dying cells per tissue in genotypes shown in (A) (n=17, number of brains), (B) (n=17), (C) (n=17, number of midguts), (D) (n=13), (E) (n=19, number of histoblasts), and (F) (n=11). Error bars, SEM; n.s, not significant; non-parametric Mann-Whitney *U*-test.

Genotypes are as follows: *tub-Gal4/UAS-CD8-PARP-Venus* (A), *tub-Gal4/UAS-CD8-PARP-Venus, RpS3^{Plac92}* (B), *esg^{NP5130}*/+; *UAS-CD8-PARP-Venus*/+ (C, E), and *esg^{NP5130}*/+; *UAS-CD8-PARP-Venus, RpS3^{Plac92}*/+ (D, F).

For the rescue experiments shown in Fig 4C, the authors find that heterozygosity for dilp8 partially overcomes the developmental delay produced by heterozygosity for RpS3 as does nub>dilp8 RNAi. However, the rescue observed when they feed ecdysone (Fig. 4E) is much better (less delay). Is this difference simply because dilp8 expression was not fully attenuated, or are there other signals involved? What happens to the delay if dilp8 RNAi is ubiquitously expressed?

Response:

As pointed out by the reviewer, heterozygosity for *dilp8* or *nub>dilp8* RNAi only partially rescued the developmental delay of *RpS3/+* animals, which could be because *dilp8* expression was not fully attenuated in these experimental settings or other tissues also contribute to *dilp8* upregulation. Indeed, we have now found that the number of dying cells was also significantly increased in other tissues such as eye discs, leg discs, and haltere discs in *RpS3/+* larvae compared to wild type larvae (see Fig R5, below), although it was not as drastic as the increase in the wing discs. In addition, *dilp8-GFP* expression was upregulated in these discs in *RpS3/+* larvae (see Fig R6, below).

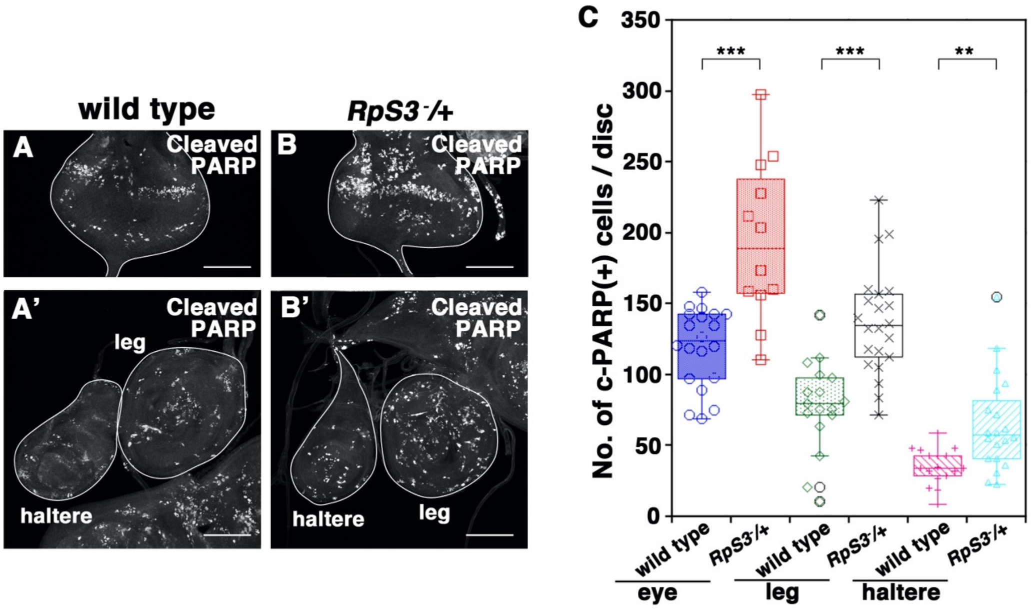

Fig. R5.

(A-B) Dying cells were detected by anti-cleaved PARP staining (white) in the eye discs, leg discs, or haltere discs of *tubulin-Gal4*(A), or *RpS3*/+, *tubulin-Gal4* (B) flies expressing CD8-PARP-Venus. Scale bar, 100 μm.

(C) Boxplot with dots representing cleaved-PARP-positive dying cells per tissue in genotypes shown in (A) (n=20, number of eye discs), (B) (n=12, number of eye discs), (A') (n=17, number of leg discs; n=17, number of haltere discs), and (B') (n=22, number of eye discs; n=20, number of haltere discs). Error bars, SEM; Error bars, SEM; ***, p<0.001, **, p<0.01; non-parametric Mann-Whitney *U*-test.

Genotypes are as follows: *tubulin-Gal4/UAS-CD8-PARP-Venus* (A), and *tubulin-Gal4/UAS-CD8-PARP-Venus, RpS3*^{Plac92} (B).

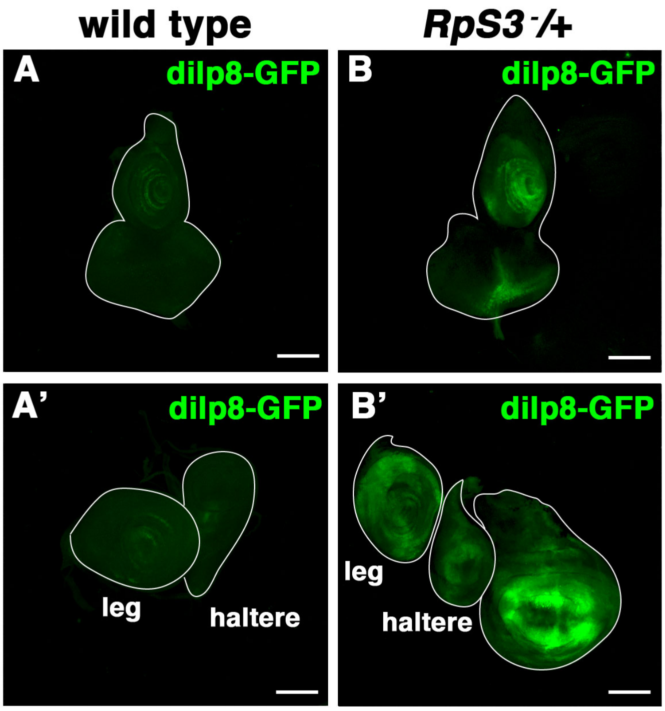

Fig. R6.

(A-C) The expression of dilp8 in eye discs (A, B), leg discs, or haltere discs (A', B') of *dilp8::eGFP/+* (A), or *RpS3/+, dilp8::eGFP/+* (B) flies were visualized with GFP (green). Scale bar, 100 μm.

Genotypes are as follows: *dilp8::eGFP/+* (A) and *dilp8::eGFP/RpS3^{Plac92}* (B).

These data suggest that other tissues also contribute to *dilp8* upregulation in *RpS3/+* larvae. To address this, we have performed ubiquitous overexpression of *dilp8-RNAi* in *RpS3/+* animals using the *tubulin-Gal4* driver. Unfortunately, however, *RpS3/+* animals ubiquitously expressing *dilp8-RNAi* resulted in larval lethal at around 100 hrs AEL (early 3rd instar), which precluded us to measure the larval period in these animals.

Minor point: line 244 "…found that the number of cell death…" please reword

Response:
We have now reworded as "the number of dying cells".

Reviewer #3:

In this manuscript, the authors examine the Minute (M/+) phenotype in more detail using wing imaginal discs in Drosophila. M mutations affect ribosomal genes and cause a significant developmental delay. It was assumed that this delay is caused by reduced proliferation rates due to decreased ribosomal activity. However, contrary to this assumption, the authors found that at least one M mutation (RpS3-/+) is characterized by increased proliferation rates. Likewise, the authors observed significantly increased apoptosis in this and two other M/+ mutants. The authors then demonstrate that M/+ wing pouches exhibit greatly enhanced cell turnover that is induced by massive apoptosis and subsequent compensatory proliferation. In search for the apoptosis-inducing signal, the authors found that increased expression of Wg in an aberrant (steeper) gradient in the wing disc triggers apoptosis in M/+ discs. However, the steeper Wg gradient alone is not sufficient for apoptosis. The authors further found that the developmental delay of M/+ mutants contributes to the aberrant Wg gradient and apoptosis. Consistently, down-regulation of dilp8 which promotes the developmental delay, reduces the amount of apoptosis in M/+ discs. In addition, feeding ecdysone (E20) to M/+ larvae rescues the developmental delay and apoptosis. Finally, overexpression of wg under wg control which in wild-type larvae has very little effect

on apoptosis, triggers strong apoptosis in ecdysoneless mutant larvae which are developmentally delayed independently of M/+.

This is a very interesting manuscript. It reports the very surprising discovery that M/+ wing discs are characterized by massive cell turnover due to increased apoptosis and compensatory proliferation. This cell turnover is dependent on the developmental delay and ensures developmental robustness in M/+ mutants. The data are very convincing and often different methods were applied to ensure consistency. The manuscript is very well written. I do have a few experimental suggestions and comments/questions to further improve the manuscript.

1. It would be good if the authors can show the increased proliferation in more than one M/+ mutant. They showed the increased cell death for 3 M/+ strains, but the proliferation phenotype was only shown for one.

Response:

We thank the reviewer for the comment. Following the reviewer's suggestion, we have now examined whether other *M*/+ strains also exhibit increased proliferation in the wing pouch. We found that cell proliferation was indeed significantly increased in the pouches of *RpS17*/+ and *RpL19*/+ larvae, as visualized by the M phase marker anti-phospho-Histone H3. We have now included these new data in the revised manuscript as Fig S1A-S1C as follows:

(page 5, paragraph 1)

"Similar increased cell proliferation was also observed in the wing pouches of other *M/*+ strains such as *RpS17/*+ and *RpL19/*+ (S1A and S1B Figs, quantified in S1C Fig)."

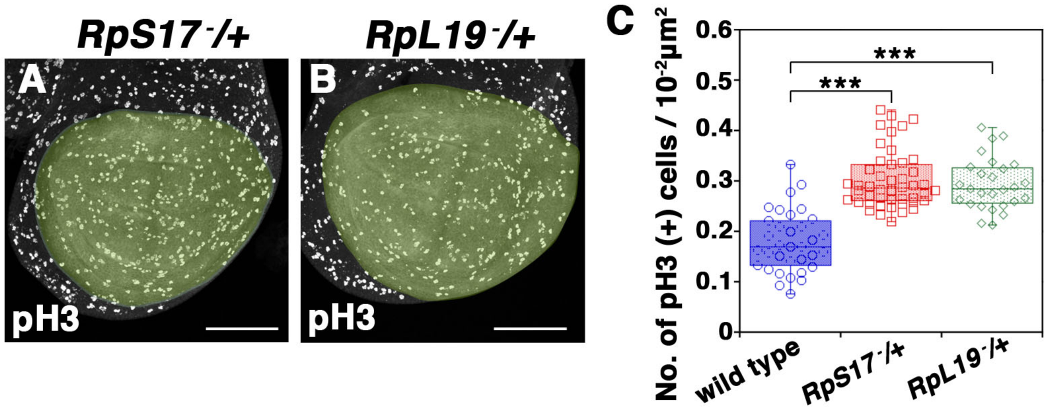

S1 Fig.

(A and B) Wing disc of *RpS17/+* (A) or *RpL19/+* (B) flies were stained with anti-phospho-histone H3 (pH3) (Ser10) antibody (white). Wing pouches were marked by pale green. Scale bar, 100 μm.

(C) Boxplot with dots representing pH3 positive cells in the pouch in genotypes shown in (Fig 1A) (n=34, number of wing pouches), (A) (n=46) and (B) (n=27). Error bars, SEM; ***, p<0.001; non-parametric Mann-Whitney *U*-test.

Genotypes are as follows: *nub-Gal4/+; UAS-CD8-PARP-Venus/RpS17*^{4} (A, D), *nub-Gal4/RpL19*^{k03704}*; UAS-CD8-PARP-Venus/+* (B, E),

2. If I understand this correctly, the aberrant steeper Wg gradient is the result of the continuous wg expression during the delay period in M/+ wing discs. If correct, it would follow that the massive apoptosis is only triggered during the period of the developmental delay (so quite late in larval development). However, the authors write that “cell death increased as development preceded and peaked during middle to late 3rd instar” (lines 117-118). Is that consistent with the dependence of apoptosis on the aberrant Wg gradient and the developmental delay? When do the authors detect the aberrant Wg gradient first in development?

<u>Response:</u>

We have shown that the aberrant Wg signaling activity is caused by the combination of elevated Wg expression (Figs S3A and S3D) and developmental delay in *M*/+ animals. Following the reviewer’s comment, we have now examined the Wg signaling activity using the *nmo-LacZ* reporter during the larval development of *RpS3/+* animals with a genetic rescue of RpS3 protein in the posterior compartment. We found that aberrant Wg signaling activity was detected at around 98 hrs after egg laying (see Fig. R7 below, please compare the LacZ staining patterns in Fig. R7C and Fig. 3E, F). Given that massive cell death occurs at around 130 hrs AEL (Fig. S1G), it is considered that aberrant Wg signaling activity and developmental delay cooperate to induce massive cell death at this period.

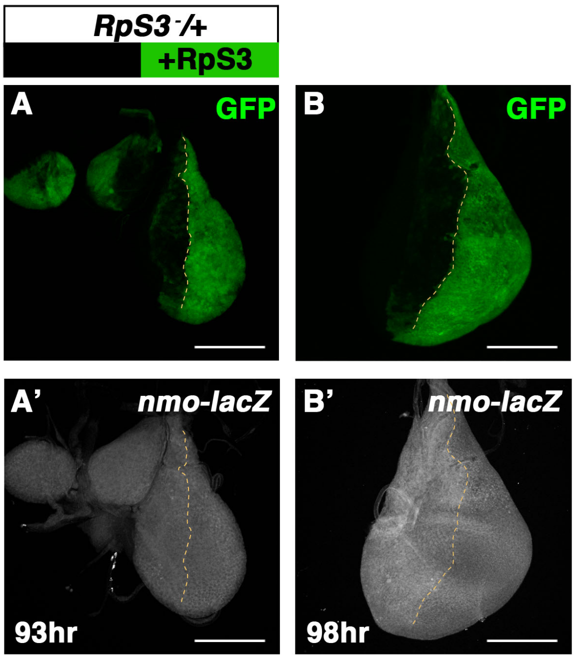

Fig. R7

(A-B’) RpS3 was overexpressed in the posterior compartment of wing discs of *RpS3/+, nmo-lacZ/+* flies using the *en-Gal4* driver at the 93hr (A), or 98hr (B) after egg laying of 3rd instar larvae. *nmo* expression was visualized by anti-β-galactosidase staining (white). Scale bar, 100 μm.

Genotype is as follows: *en-Gal4, UAS-GFP/+; RpS3*^{Plac92}*, UAS-RpS3/nmo*^{P1} (A-B’).

3. Wg expression along the DV border is dependent on Notch signaling. Did the authors observe any abnormalities of Notch signaling in M/+ discs?

Response:

Following the reviewer’s suggestion, we have examined whether Notch signaling is affected in the *M*/+ wing discs using the NRE-EGFP reporter that visualizes Su(H)-dependent Notch signaling (Saj *et al*., *Dev Cell*., 2010). We found that Notch signaling is indeed elevated in *RpS3*/+ wing pouch, as genetic rescue of RpS3 in the posterior compartment of the *RpS3*/+ wing disc resulted in decreased Notch signaling activity compared to the anterior control, while overexpression of RpS3 alone did not affect Notch signaling activity in the wild-type wing disc (see Fig. R8, below). These data suggest the possibility that increased Notch signaling activity causes Wg upregulation in *M*/+ wing discs. We would like to further investigate the role and mechanism of Notch signaling activation in *RpS3*/+ wing pouch in the future study.

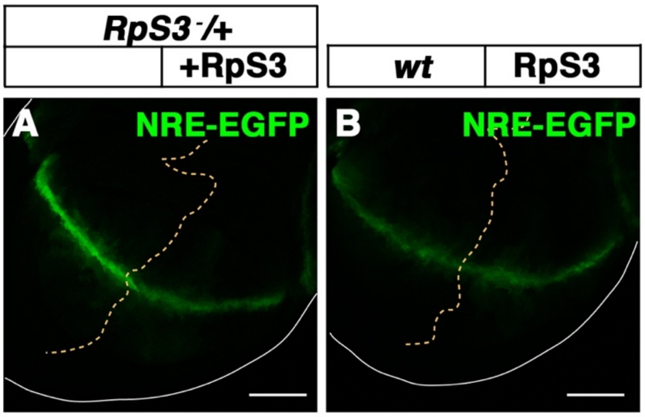

Fig. R8

(A and B) RpS3 was overexpressed in the posterior compartment of the wing discs of *RpS3*/+ (A) or wild-type (B) flies using the *en-Gal4* driver. Su(H)-dependent Notch signaling was visualized by NRE-EGFP reporter (green). Scale bar, 100 μm.

Genotypes are as follows: *en-Gal4.UAS-RFP/NRE-EGFP; UAS-RpS3, RpS3*^{Plac92}/+ (A), and *en-Gal4.UAS-RFP/NRE-EGFP; UAS-RpS3*/+ (B).

4. Is the enhanced cell turnover also observed in other discs such as eye discs? If so, would there be a similar dependence on Wg in this tissue?

Response:

Following the reviewer's suggestion, we have now quantitatively analyzed cell death in other tissues in *RpS3*/+ larvae. As a result, we found that the number of dying cells was significantly increased in the eye discs, leg discs, and haltere discs as well in *RpS3*/+ animals compared to wild type (see Fig. R9, below). However, the increase in the number of dying cells in these tissues (1.5-2 fold) was much less than that in the wing discs (~10 fold). In addition, we did not observe any specific pattern of dying cells as was seen in the wing discs. These data suggest that the increased cell death in these tissues could be caused by other mechanisms (e. g., simply by a developmental delay). We would like to investigate the mechanism of cell death induction in these tissues in the future study.

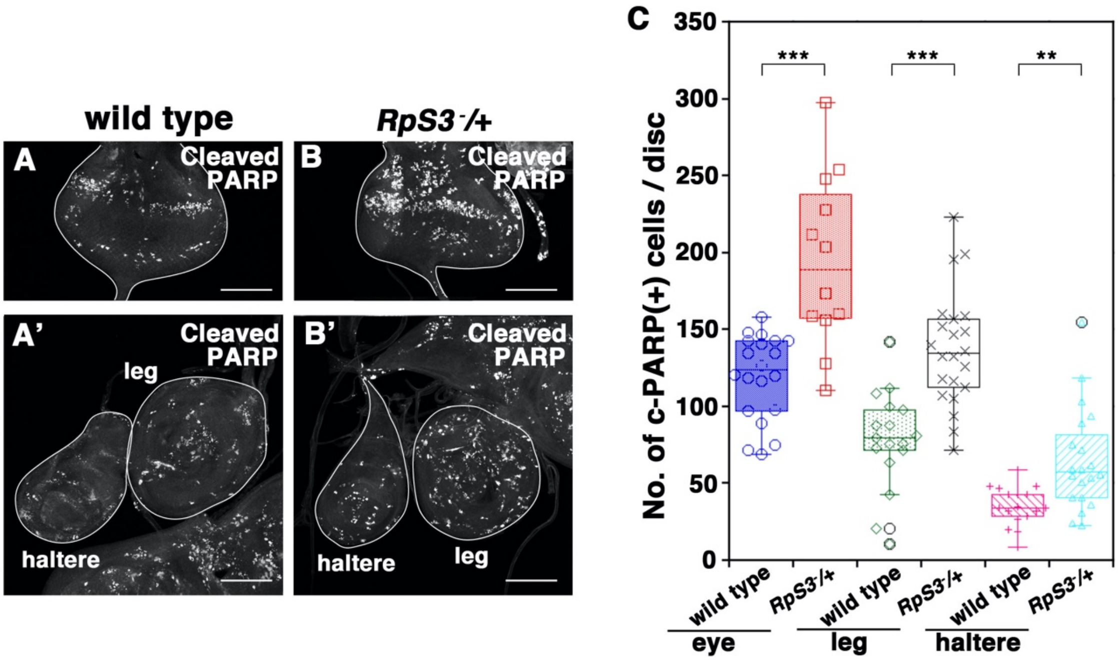

Fig. R9.

(A-B') Dying cells were detected by anti-cleaved PARP staining (white) in the eye discs, leg discs, or haltere discs of *tubulin-Gal4*(A), or *RpS3*/+, *tubulin-Gal4* (B) flies expressing CD8-PARP-Venus. Scale bar, 100 μm.

(C) Boxplot with dots representing cleaved-PARP-positive dying cells per tissue in genotypes shown in (A) (n=20, number of eye discs), (B) (n=12, number of eye discs), (A') (n=17, number of leg discs; n=17, number of haltere discs), and (B') (n=22, number of leg discs; n=20, number of haltere discs). Error bars, SEM; Error bars, SEM; ***, p<0.001; **, p<0.01; non-parametric Mann-Whitney *U*-test.

Genotypes are as follows: *tubulin-Gal4/UAS-CD8-PARP-Venus* (A), and *tubulin-Gal4/UAS-CD8-PARP-Venus, RpS3*^{Plac92} (B).

5. In the ecd;wg>wg experiment where the authors detect increased apoptosis (Fig. 4N), is there also compensatory proliferation?

Response:

We thank the reviewer for the comment. We have now examined whether compensatory proliferation also occurs in *ecd/ecd* flies overexpressing Wg by the *wg-Gal4* driver. We found that cell proliferation was indeed elevated in the wing pouches of these flies. We have now included these new data in the revised manuscript as S4 Fig as follows:

(page 9, paragraph 3)

"Strikingly, a forced increase in endogenous Wg expression in conjunction with developmental delay caused massive cell death in the wing pouch along the DV axis (Fig 4N, quantified in Fig 4O), which was accompanied by increased cell mitoses (S4I and S4J Figs, quantified in S4K Fig)."

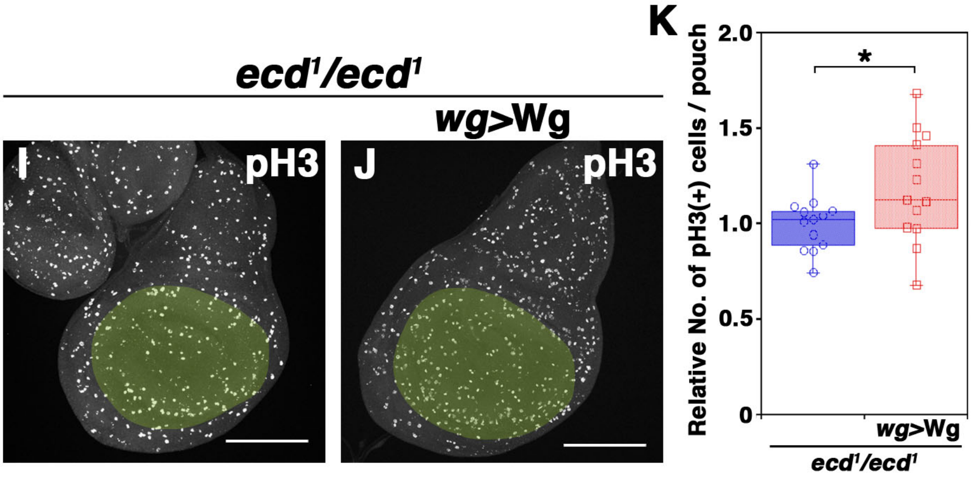

S4 Fig.

(I and J) Wing disc of *ecd*^{1}/*ecd*^{1} (I) or *ecd*^{1}/*ecd*^{1}, *wg-Gal4,* UAS-Wg (J) were stained with anti-phospho-histone H3 (pH3) (Ser10) antibody (white). Wing pouches are marked by pale green. *ecd*^{1} is a temperature-sensitive *ecd* mutant allele that blocks biosynthesis of the active-form of the hormone 20-Hydroxyecdysone at 29˚C. For heat-shock treatment, fly culture was transferred to 29˚C for 48 hours during the 3rd instar 1arval stage. Scale bar, 100 μm.

(K) Boxplot with dots representing pH3 positive cells per pouch in genotypes shown in (I) (n=13, number of wing pouches) and (J) (n=13). Error bars, SEM; *, p<0.05; non-parametric Mann-Whitney *U*-test.

Genotypes are as follows: *ecd*^{1}/*ecd*^{1} (A, L), *wg-Gal4/UAS-Wg; ecd*^{1}/ *ecd*^{1} (B, M),

6. Explain in the manuscript, what the erg-2 mutant yeast in the fly food accomplishes.

Response:

Following the reviewer's suggestion, we have now added explanation for what the erg-2 mutant yeast accomplishes in the fly food in the 'Method' section in the revised

manuscript as follows:

(page 15, paragraph 2)

"Fly cultures with ecdysteroid synthesis defect

To inhibit the ecdysteroid synthesis, larvae were reared in the food with *erg-2* sterol mutant strain (a gift from Tomonori Katsuyama). Given that the *erg-2* gene encodes the Δ8-Δ7-sterol isomerase, flies feeding food with *erg-2* yeast impair production of a sufficient titer of ecdysteroid hormone necessary for pupation, leading to the developmental arrest in the larval stage. To collect embryos, flies were allowed to lay eggs for 8 hours at 25°C on acetic acid agar plates. Embryos were rinsed, dechorinated with 3% bleach, and rewashed with MilliQ. Embryos were then put on a cover glass and transferred into *erg-2* Δ yeast food."
